# Safe and Efficient Sigma1 Ligand: A Potential Drug Candidate for Multiple Sclerosis

**DOI:** 10.3390/ijms231911893

**Published:** 2022-10-06

**Authors:** Bénédicte Oxombre, Fahima Madouri, Anne-Sophie Journé, Séverine Ravez, Eloise Woitrain, Pascal Odou, Nathalie Duhal, Sandro Ninni, David Montaigne, Nadira Delhem, Patrick Vermersch, Patricia Melnyk

**Affiliations:** 1 U1172—LilNCog—Lille Neurosciences & Cognition, Université de Lille, Inserm, CHU Lille, F-59000 Lille, France; 2 SATT Nord, F-59800 Lille, France; 3 U1011—Récepteurs Nucléaires, Maladies Métaboliques et Cardiovasculaires, Université de Lille, Inserm, CHU Lille, F-59000 Lille, France; 4 ULR 7365—GRITA—Groupe de Recherche sur les Formes Injectables et les Technologies Associées, Université de Lille, CHU Lille, F-59006 Lille, France; 5 CUMA—Centre Universitaire de Mesures et d’Analyses, Université de Lille, F-59000 Lille, France; 6 CHU Lille, UMR 1189—OncoThAI (Thérapies Assistées par Lasers et Immunothérapies pour l’Oncologie), Université de Lille, Inserm, F-59000 Lille, France; 7 Immune Insight, Institut de Virologie de Lille, F-59021 Lille, France

**Keywords:** benzamide, sigma1 receptor, CNS, multiple sclerosis

## Abstract

Multiple Sclerosis (MS) is an autoimmune demyelinating and neurodegenerative disease of the central nervous system (CNS). Current management strategies suppress or modulate immune function, all with consequences and known side effects. They demonstrate a high level of success in limiting new relapses. However, the neurodegenerative process still affects both grey and white matter in the central nervous system. The sigma1 (S1R) ligand-regulated chaperone is implicated in many biological processes in various CNS-targeted diseases, acting on neural plasticity, myelination and neuroinflammation. Among the proteins involved in MS, S1R has therefore emerged as a promising new target. Standard and robust methods have been adopted to analyze the adsorption, distribution, metabolism, excretion (ADME) properties, safety pharmacology and toxicology of a previously synthetized simple benzamide-derived compound with nanomolar affinity for S1R, high selectivity, no cytotoxicity and good metabolic stability. The compound was also characterized as an agonist based on well-validated assays prior to in vivo investigations. Interestingly, we found that the oral administration of this compound resulted in an overall significant reduction in clinical progression in an MS experimental model. This effect is mediated through S1R action. Our results further suggest the potential use of this compound in the treatment of MS.

## 1. Introduction

New drugs targeting the central nervous system (CNS) have been increasingly studied and characterized over the span of the last half-century. One of the biggest challenges faced in preclinical trials of CNS-targeted disease is that drugs have to cross through the blood brain barrier (BBB). It is also mandatory to establish an appropriate balance between drug efficacy and potential adverse effects in the early stages of evaluation. In Alzheimer’s disease (AD), the ABCDE paradigm has been recently defined as a conceptual tool to facilitate the development of safe and effective therapies that can be summed up as being (A) accessible, (B) BBB permeant, (C) associated with improvements in clinical symptoms, (D) disease-modifying and (E) environmentally nontoxic [1]. This ABCDE concept is in fact applicable to all CNS diseases. The chemical structure of a drug is indicative of its physicochemical properties (solubility, stability, etc.), and its drug-like properties such as adsorption, distribution, metabolism, excretion (ADME) and toxicity, also including metabolic stability, plasma stability, P-glycoprotein (P-gp) extrusion, serum albumin binding, cytochrome P450 (CYP) inhibition or human Ether-à-go-go Related Gene (hERG) inhibition. These characteristics affect drug bioavailability and pharmacodynamic activity, which forecast its potential clinical achievements. Preclinical studies essentially enable us to define these parameters and assess whether a new drug could be a moderate or even low risk candidate while being considered for entering a clinical trial.

Multiple sclerosis (MS) is the leading cause of non-traumatic neurological disability arising in young adults. MS is characterized by the pathological hallmarks of inflammation with demyelination, astrogliosis and neurodegeneration. Tissue damage in MS is restricted to the CNS and MS symptoms vary according to the location and severity of the lesions. MS can also present a relapsing or progressive evolution, but the same underlying disease process remains engaged. Inflammation is typically associated with relapses while neurodegeneration is associated with the progression of MS. Current therapeutic strategies are focused on managing the inflammatory component. Several monoclonal antibody therapies have been developed in the past decade [2]. B-cell depleting strategies demonstrate high success in limiting new relapses and the accumulation of focal lesions using magnetic resonance imaging. The impact of currently approved drugs is limited on disability in the progressive forms of MS [3]. Despite these drugs exhibiting many beneficial effects, some serious adverse effects (AE) have been reported in many experiments and clinical trials [4] and reparative approaches are still needed. In addition, biologics are relatively complex molecules to develop. One current research strategy is the development of small molecule drugs that can be administered by a variety of routes, especially orally [5]. The neurodegenerative process occurs early on in both gray and white matter of the CNS, and may clearly play a significant role in the first stages of MS. Mitochondrial dysfunction is considered to be one of the main contributors to axonal damage [6]. In fact, mitochondrial homeostasis is essential in maintaining proper energy production and calcium regulation, both of which are crucial functions in the healthy functioning of neurons and oligodendrocytes.

The sigma opioid receptor was described for the first time in 1976 by Martin et al. but later recognized as sigma receptor by Su TP [7,8]. The two subtypes of sigma receptors were identified as sigma1 (S1R) and sigma2 (S2R) receptors, and S1R was successfully cloned in 1996 [9]. Anatomically, S1R is expressed in peripheral organs such as the kidney, liver and lung, but our interest resides in the fact that S1R is widely distributed in numerous brain areas. Numerous studies indicate the role of S1R in neurological disorders such as schizophrenia, depression and anxiety, addiction and alcoholism, as well as in neurodegenerative disorders [10,11,12]. Cellular studies have shown that S1R is located on the endoplasmic reticulum (ER)/mitochondria interface, in a region called the mitochondria-associated ER membrane (MAM), and that it is implicated in proper Ca^2+^ signaling between the ER and mitochondria [13,14]. Under normal resting conditions, S1R forms a complex with another chaperone, the glucose-regulated protein GRP78, also known as BiP. Under pathological/stressful conditions, the Ca^2+^ concentration at the ER dramatically drops and S1R dissociates from BiP. Studies also indicate that S1R translocates to other subcellular compartments when cells are stimulated or when undergoing prolonged stress. Functionally, S1R is not a typical pharmacological receptor, as no transduction systems have been identified [15]. S1R has been characterized as a unique ligand-operated molecular chaperone [16] that can amplify or reduce the signaling incurred upon activation of several receptors, ion channels, transporters and enzymes, and thus is involved in the modulation of different physiological functions [17,18]. As a ligand-regulated chaperone, the modulatory activity of S1R can be augmented or inhibited. S1R binds diverse classes of pharmacological ligands. Some of the major high-affinity S1R ligands function as pharmacological agonists or antagonists and result in physiological response. There are also allosteric modulatory ligands that have no pharmacological activities by themselves but which elicit a positive or negative modulatory effects when certain physiological processes are engaged [19]. It must also be considered that S1R does not evoke an effect under normal physiological conditions, but results in marked cellular effects caused by being activated in stressful conditions [20,21]. In the CNS, S1R contributes to many biological processes and S1R compounds may offer promise as a disease-modifying pharmacological target in various CNS-targeted diseases by acting on neural plasticity, myelination and neuroinflammation [11,22,23]. Among the proteins involved in MS, S1R has therefore emerged as a promising new target [12,24].

Since its discovery, significant progress in the understanding of S1R function has been achieved. Pharmacophore modeling and crystal structures have provided knowledge essential in understanding its modulation. Extensive drug discovery campaigns have provided the development of small chemical molecules targeting S1R [25]. In our laboratory, tetrahydroisoquinoline-hydantoin has been described as a key pharmacophore [26] and our previous study evidenced that a single injection of one high-affinity and selective S1R agonist with a tetrahydroisoquinoline-hydantoin scaffold reduces the clinical progression of disease. This has been observed in an MS experimental model through the prevention of mononuclear cell accumulation and demyelination in the brain and spinal cord [27]. Nevertheless, this compound showed low metabolic stability. Therefore, a novel series of benzamide-derived compounds were designed, synthetized and pharmacologically evaluated [28]. Many S1R ligands have mixed binding affinity for both S1R and S2R, as well as other receptor types; however, compound **7i** showed excellent affinity for S1R (Ki = 3.2 nM) and selectivity for S2R (Ki up to 1400 nM) with a high selectivity index (Table 1 and Table 2). Undesirable off-target effects were identified early using pharmacological profiling against 40 different receptors relevant to CNS disease, which indicated that compound **7i** had an excellent highly selective profile. Compound **7i** also presented no cytotoxicity. It is also interesting because the physical properties of compound **7i** are also favorable for drug development.

In the present study, an extensive in vitro analysis was used in order to establish as many arguments as possible to show that compound **7i** exhibits ideal characteristics that allow it to be considered a good drug candidate for CNS-targeted diseases. The impact of compound **7i** was then analyzed in vivo to validate that the new drug presents a moderate risk for clinical application and that S1R modulation significantly reduces clinical progression of the disease in an MS experimental model.

## 2. Results

### 2.1. In Vitro Drug Candidate Assessment

#### 2.1.1. Metabolic Stability and Metabolite Identification and Profiling

Kinetic study of the microsomal stability of compound **7i** was performed in mouse and human liver microsomes (i.e., SER membrane re-form into vesicles) at 10 µM (Table 1). Compound **7i** shows a half-life greater than 1 h and exhibits good clearance (<115 µL/min/pmol). After 1-h incubation, only 20% of compound **7i** is metabolized and detected in demethylated form (named hereafter as **7i-deMe**, Figure S1). Demethylation slightly decreases S1R affinity (23 nM), and more drastically S2R affinity (>1000 nM), increasing the S2R/S1R ratio by up to 500 (Table 2). The potential cytotoxic effects of compound **7i-deMe** were analyzed on a human neuroblastoma cell line at different concentrations up to 100 µM. Compound **7i-deMe** exhibits higher cytotoxicity than compound **7i** [28]. Compound **7i-deMe** shows a half-life greater than 3 h in mouse liver microsomes and 9 h in human liver microsomes (Table 1). No modification of clearance is observed. Its affinity for S1R, its selectivity and its low cytotoxicity make it a compound comparable to compound **7i**.

#### 2.1.2. Preliminary ADME Studies

Compound **7i** was then evaluated for in vitro properties essential for later stages of drug development. In the first step, preliminary ADME study was focused on bioavailability features at 10 µM (Table 3). As expected, compound **7i** exhibited high solubility in PBS solution at pH 7.4 (185 µM). Solubility and stability were not modified using simulated gastric fluid (SGF) and intestinal fluid (SIF), mimicking digestion. Permeability across cell membranes was assessed to investigate intestinal permeability and predict the absorption of orally administered drugs using apical (A, pH 6.5) and basolateral (B, pH 7.4) chambers representing the luminal and blood/mesenteric lymph sides of the gastrointestinal tract, respectively. Compound **7i** shows good A/B permeability (up to 20 × 10^−6^ cm/s) and an efflux ratio close to 2. Its stability in plasma lasts greater than 24 h. Compound **7i** affinity to plasma protein is below 95%. In the liver, CYP enzymes are important metabolic players. At the biochemical level, CYP families 1 to 3 constitute major enzymes for the biotransformation of small molecule drugs [29]. Therefore, CYP inhibition analysis was performed on human 1A, 2B6, 2C8, 2C9, 2C19, 2D6 and 3A as recommended by FDA and EMA guidance. No to weak inhibition was observed, except for CYP2D6. Membrane transport proteins can also influence the pharmacokinetics of many drugs and may be implicated in drug–drug interactions. They are categorized into ATP-binding cassette (ABC) and solute-linked carrier (SLC) families and are expressed by several tissues, such as the intestine, liver, kidney and brain [30]. In a third step, the inhibitory effects of compound **7i** were evaluated in five ABC (P-gp, BCRP, MRP1, 2 and 3) and eight SLC (OATP1B1 and 3, OAT1 and 3, OCT1 and 2, ABST and NTCP) transporters. The inhibitory effects of compound **7i** are low (highest values are below 50% inhibitory effect), showing that the compound has no membrane transporter inhibitor capacity. Membrane transporters also play a critical role in new molecular entity (NME) absorption through active transport. Metabolite **7i-deMe** has similar characteristics to compound **7i** (Table 3).

P-gp was identified as a major barrier protein for many CNS drug substances, and P-gp substrate activity remains a key selection target for CNS drug discovery [30]. Comparison of efflux ratios generated in the presence or absence of the P-gp inhibitor verapamil at pH 7.4 (0.3 and 0.6, respectively) indicates that compound **7i** is not a substrate for P-gp transporter. Induction of the expression of CYPs can also dramatically alter the pharmacokinetics of a drug, its toxicity and impact drug–drug interactions [31]. CYP induction was evaluated on human hepatocytes (three donors). No modulation of CYP1A2, CYP2B6 or CYP3A4 was observed at 10 µM.

#### 2.1.3. Safety Pharmacology and Toxicology

In vitro pharmacology profiling is increasingly being used earlier in drug discovery processes to identify undesirable off-target activity profiles that could hinder or halt the development of candidate drugs. Cardiac toxicity is one of the major reasons for drug failure. Early robust profiling panels are based on interaction with the well-characterized cardiac ion channel. Analysis was performed at three regulatory concentrations on the potassium voltage-gated channel subfamily H member 2 (KCNH2; also known as hERG) that is the only in vitro pharmacology assay absolutely required by regulatory authorities to study the effects of new chemical entities on ionic current [32]. Compound **7i** was shown to inhibit hERG with an IC_50_ of 1 µM. Comparable results were obtained with compound **7i-deMe** (IC_50_ of 1.2 µM). The cytotoxicity analysis of compound **7i** was thorough and used human hepatocytes HepG2 cells (Table 4). No modifications in cell number, nuclear size, mitochondrial membrane potential, intracellular free calcium or membrane permeability were observed. Genotoxicity testing of drug candidates is also required to support clinical entry. The most common approach is to perform two separate in vitro assays (one by a bacterial reverse mutation and one in a mammalian cell) before any in vivo analysis. For in vitro mammalian cell testing, the micronucleus test (MN) is commonly used [33]. Compound **7i** has no mutagenic effects, as shown by Ames tests on five bacterial strains (Table 5). The same results were obtained at six increasing concentrations (0.37 to 40 µg/plate). Table 6 shows the MN/2000-nuclei ratio and relative survival (RS) obtained in TK6 cells treated with compound **7i** in two independent experiments. Cells treated with the positive controls show a significant increase in MN compared to the vehicle control cultures. Compound **7i** shows no genotoxic characteristics.

**Figure 1:**
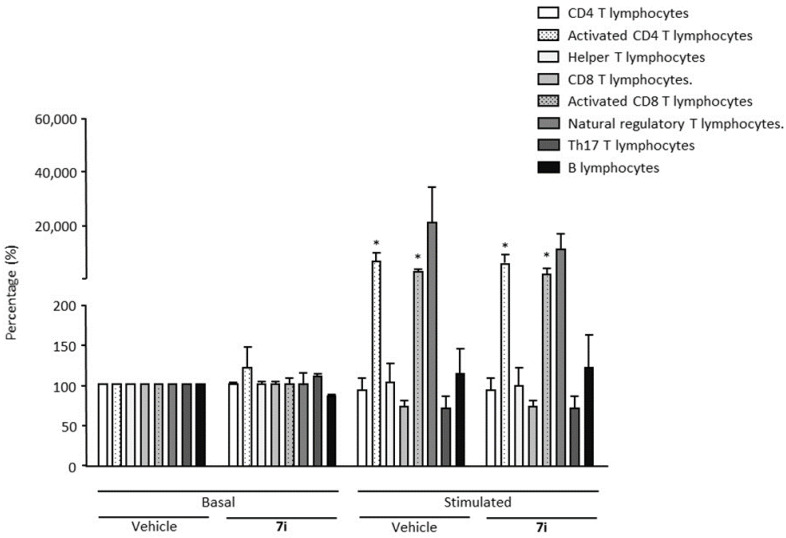
Immunotoxicity analysis. Human peripheral blood mononuclear cells (hPBMCs) were either non-stimulated (basal) or stimulated during the course of a 48-h period. Compound **7i** was tested at 10 µM. Immune cell subpopulations were immunophenotyped among hPBMCs by flow cytometry. Cell percentage was evaluated. Data are presented as mean ± SEM from six individual healthy donors. * *p* < 0.05 vs. control (basal vehicle) condition. Wilcoxon–Mann–Whitney U test.

Chronic inflammation contributes to numerous diseases. Although it is not required to perform immunotoxicity assays prior to standard toxicology studies (STS) testing, we decided to analyze the impact of compound **7i** on human peripheral blood mononuclear cells (hPBMCs) that were or were not stimulated with *E. coli* lipopolysaccharide (LPS) and αCD3/αCD28 for specific TCR activation. Lymphocyte subpopulations were immunophenotyped, including CD19 B, CD4 and CD8 T lymphocytes and natural regulatory T cells (Figure 1). On basal non-stimulating conditions, compound **7i** has no effect on the percentage of human B, Th17 and T lymphocyte subpopulations (CD4 and CD8 T cells), nor regulatory T cells. As expected, in TCR stimulating conditions, a significant increase in activated CD4 and CD8 T lymphocytes has been observed, suggesting that compound **7i** does not affect cellular immune adaptive systems and that immune function is preserved. This was confirmed by natural regulatory T cell prevalence that was also increased to naturally restore immune cell homeostasis. We also confirmed that compound **7i** has no effect on Th17 cells that are known to induce an inflammatory microenvironment. Altogether, these results suggest that compound **7i** has no toxicity effect on all immune subpopulations tested in stimulating or not stimulating conditions.

### 2.2. Evaluation of Compound ***7i*** as a Sigma1 Receptor Agonist

S1R was historically classified as a receptor because a large number of high-affinity and selective small ligands triggered (for so-called agonists) or prevented (for so-called antagonists) biological responses. This understanding remains despite the fact that S1R’s mode of action relies on the modification of protein–protein interactions rather than coupling to second messenger systems, suggesting more of a chaperone-like identity than that of the classical nature of a receptor [14].

**Figure 2:**
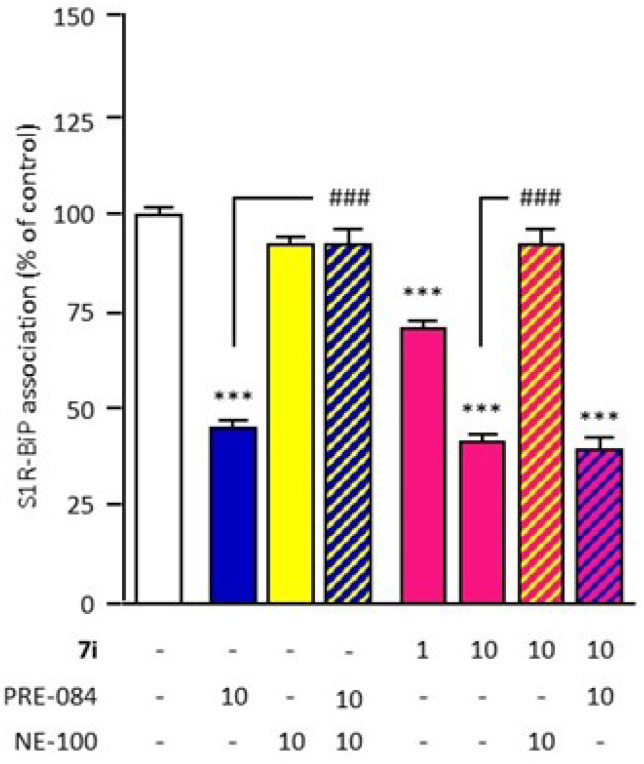
In vitro compound **7i** agonist activity evaluation. The effect of compound **7i** (1 and 10 µM) on S1R–BiP association (30-min incubation) was analyzed through co-immunoprecipitation with an S1R antibody (red). BiP level was measured using ELISA. PRE−084 (blue) and NE−100 (yellow) were used for agonist and antagonist of reference, respectively (10 µM). Agonist activity was validated by blockade by NE−100 (yellow hatching). The absence of antagonist activity was validated with PRE−084 (blue hatching). Data are presented as mean ± SEM of *n* = 6 mice per condition. *** *p* < 0.0001 vs. non treated group, ^###^ *p* < 0.0001 vs. PRE-084 or **7i** 10 μM group. Dunnett’s test.

It is described that S1R agonists promote S1R dissociation from the endoplasmic reticulum binding immunoglobulin protein (BiP), resulting in S1R chaperone activity in the cells. In contrast, S1R antagonists reinforce the association, blocking the action of S1R agonists. This activity could be evaluated by the ability to modulate S1R–BiP dissociation, quantified by ELISA after immunoprecipitation [13]. Firstly, this well-validated cell-based assay was used to analyze compound **7i** for its in vitro activity. The reference agonist PRE-084 (10 µM) significantly causes the dissociation of the S1R–BiP complex, while the antagonist NE-100 (10 µM) has no effect on S1R–BiP association (Figure 2). NE-100 also blocks the action of PRE-084, validating its agonist effect. Compound **7i** (1 and 10 μM) significantly causes a dose-dependent dissociation of the S1R–BiP complex. Its effect was blocked by NE-100 (10 µM), demonstrating the agonist capacity of compound **7i**. Dissociation was also observed after co-treatment with PRE-084 and compound **7i**, showing that compound **7i** does not block the action of PRE-084 which validates the absence of antagonist capacity.

**Figure 3:**
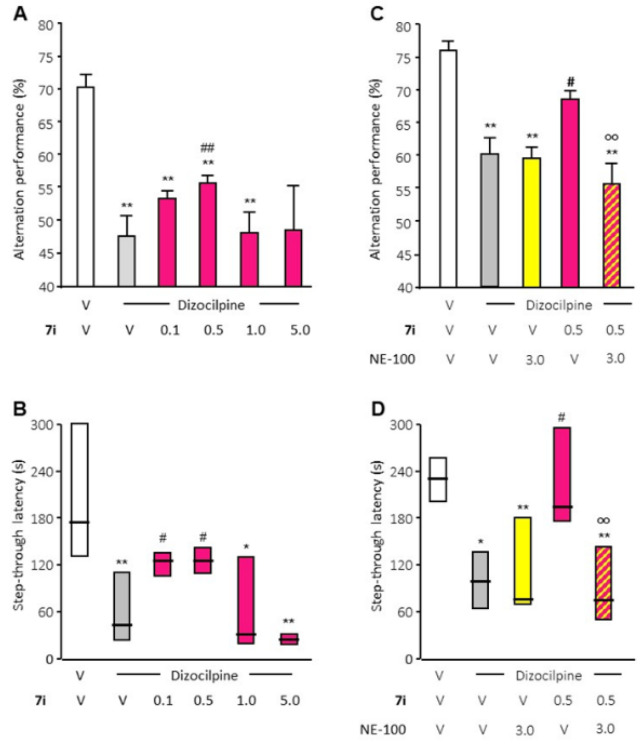
In vivo compound **7i** agonist activity evaluation. The effect of compound **7i** (0.5 to 5 mg/kg, i.p., red) was analyzed with dizocilpine-induced learning deficit analysis (V, vehicle) through spontaneous alternation performances (panels (**A**,**C**)) and passive avoidance latency (panels (**B**,**D**)). Agonist activity was validated by NE-100 blockade (3 mg/kg, i.p., yellow). Data are presented as mean ± SEM of *n* = 6 mice per condition. * *p* < 0.05, ** *p* < 0.01 vs. (vehicle + vehicle)-treated group, # *p* < 0.05, ## *p* < 0.01 vs. (Dizocilpine + vehicle)-treated group, oo *p* < 0.01 vs. (Dizocilpine + **7i**)-treated group. Dunn’s test.

Secondly, two well-described behavioral tests, validated for the evaluation of S1R activity in vivo, were performed. Compound **7i** significantly attenuated dizocilpine-induced learning deficits at 0.5 mg/kg in the Y-maze test and at 0.1 and 0.5 mg/kg in the passive avoidance test (Figure 3). The beneficial effect of compound **7i** in the two tests is prevented by treatment with the S1R antagonist NE-100 at 3 mg/kg, which is devoid of any effect by itself.

These results confirm the S1R receptor agonist effect of compound **7i**. Those in vivo behavioral tests also highlight the bi-phasic dose–response curve that is very regularly observed in the biological responses of S1R agonists [15]. The effects of compound **7i** on behavior are visible at a concentration that ranges from 0.1 to 1 mg/kg.

### 2.3. In Vivo Pharmacology

#### 2.3.1. Preliminary Pharmacokinetic Analysis

A drug candidate for CNS pathologies needs to penetrate and stay in the brain tissue for a sufficiently long time. One of the major challenges in CNS drug discovery and development is also to achieve the right balance between the free fractions in the blood and brain. In 2010, Wager et al. provided guidance for the design of successful CNS drug candidates based on several physicochemical drug properties [34]. The median optimal values were found to be partition coefficient (logP) = 2.8, molecular weight (MW) = 305.3, polar surface area (TPSA) = 44.8 Å^2^, hydrogen-bond donor (HBD) = 1 and pKa = 8.4. Compound **7i** has MW of 316 Da; logP of 3.5; TPSA of 32 Å^2^; one HBD and possesses a basic amine with a pKa value of 9.1. Therefore, compound **7i** displays desirable theoretical physicochemical properties predicting good CNS penetration. The blood–brain ratio remains a key analytical parameter that indicates the brain-targeting ability of neurotherapeutics with their brain bioavailability. It is for this purpose that the quantification of compound **7i** was performed in plasma and brain homogenate samples. As the ultimate goal of pharmacological development is therapeutic establishment, oral (p.o.) administration was prioritized. The concentration time profiles were also carried out at 1 mg/kg after intravenous (i.v.) administration in order to define the pharmacokinetic parameters of compound **7i**. In fact, the i.v. enteral route allows for relatively precise drug concentrations to be achieved in plasma since bioavailability is not a concern due to the device delivering the drug directly into the bloodstream. Conversely, in the oral parenteral route, blood draining in the gut passes through the liver, which is a major site of drug metabolism, before reaching the systemic circulation. The pharmacokinetic parameters obtained are listed in Table 7. The time needed to reach C_max_ (t_max_) was set to 0 after i.v. administration and was less than 30 min after p.o. administration (Figure S2). The maximal concentration (C_max_) in plasma after i.v. administration was determined as 180 ng/mL. Concentrations at 30 min (C_30_) after i.v. administration were more than 10 times higher than after p.o. administration, whether in the plasma or in the brain. After oral administration, compound **7i** showed a distribution volume (V_d_) higher than the total blood volume in mice (85 mL/kg), indicating extravascular distribution. Total clearance was found to be 3.8 mL/min after i.v. administration. This value was increased by 20 times when administration was performed orally. After i.v. administration, it takes 44.5 min to eliminate half of the circulating compound **7i** (t_1/2 el_). Plasma t_1/2 el_ was reduced to half 25.5 min after p.o. administration. After oral administration, the average time taken to arrive in the bloodstream (mean absorption time, MAT) was estimated as 16.5 min with 5% bioavailability. In the brain, clearance (CL_brain_) was found to be 1.8 mL/min after i.v. administration. As observed in plasma, this value was increased by 20 times after p.o. administration. Brain elimination half-life (t_1/2 el_) was estimated as 96.4 min and 78.9 min after i.v. and p.o. administration, respectively. Diffusion in the brain was estimated as 62%. A [brain]/[plasma] ratio of 3 was observed regardless of the method of administration.

**Figure 4:**
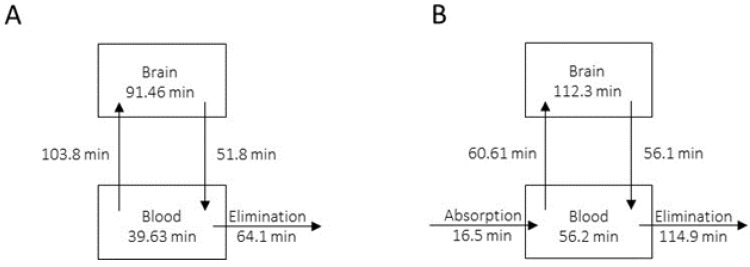
Pharmacokinetic modeling using the non-compartmental method. Compound **7i** was administrated through two methods, i.v. (panel (**A**)) and p.o. (panel (**B**)), at 1 mg/kg.

Pharmacokinetic modeling was performed using the non-compartmental method (Figure 4). Differences in time were observed and are probably the result of a limited number of individuals.

#### 2.3.2. Effects of Systemic Administration of the Selective 7i Drug on the Cardiovascular System

hERG is essential for normal electrical activity in the heart, and it has been known for a long time that arrhythmia can be induced by a blockade of this channel by a diverse group of drugs. This side effect is a common reason for drug failure in preclinical safety trials [35] because hERG channel inhibition can potentially lead to cardiac repolarization (i.e., QT interval). Electrocardiographic QT interval prolongation is the most widely used risk marker for ventricular arrhythmia potential and is thus an important component of drug cardiotoxicity assessments (Figure S3). As compound **7i** inhibits hERG in vitro at micromolar range, the effect of compound **7i** on ECG, and specifically QT interval duration was analyzed in vivo. The positive control quinidine dose-dependently prolonged the QT interval 6 to 10 min after intraperitoneal (i.p.) injection at 10 mg/kg and 100 mg/kg, while no alteration in cardiac repolarization was observed after administration of increasing concentrations (0.5 to 10 mg/kg) of compound **7i** (Table 8). In addition, the QT interval also remained stable after longer acquisition times. Furthermore, compound **7i** did not alter heart rate, nor PR or QRS duration. This present study allows us to conclude that compound **7i** does not induce a pro-arrhythmic effect.

### 2.4. In Vivo Efficacy of the Selective 7i Agonist in an MS Experimental Model

**Figure 5:**
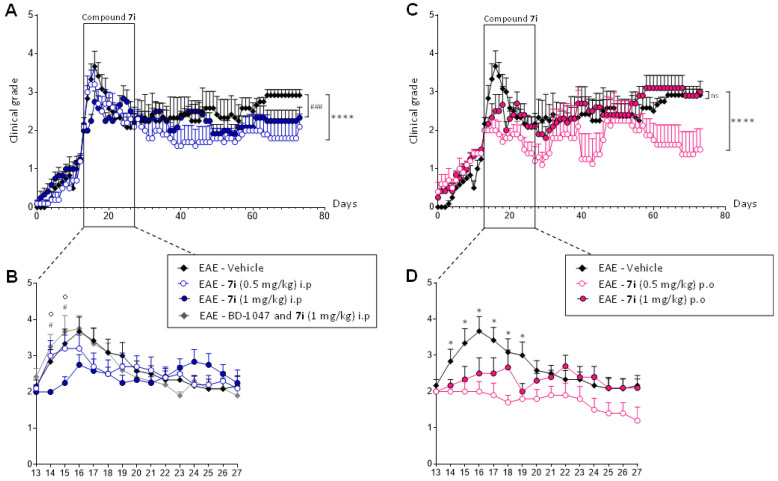
The curative effect of compound **7i** in the multiple sclerosis (MS) experimental model. Mice were immunized at day 0 (D0) and their clinical course was followed for 80 days (panels (**A**,**C**)). Compound **7i** was administrated daily for 15 days when mice reached a score of 2 (panels (**B**,**D**)). Five different groups were analyzed depending on the route of administration (i.p. or p.o.) and concentration (0.5 and 1 mg/kg). The involvement of S1R in the beneficial effect of EAE was analyzed during the first relapse (panel **B**). S1R binding sites were blocked by i.p administration of BD-1047 (10 mg/kg) 20 min before compound **7i** administration. Data are presented as mean ± SEM of *n* = 5–6 mice per condition. ns, non-significant. **** *p* < 0.0001, ### *p* < 0.001 vs. EAE—vehicle group. * or # *p* < 0.05 vs. EAE—vehicle group; o *p* < 0.05 vs. EAE—BD-1047 and **7i** group. Wilcoxon–Mann–Whitney U test.

Experimental autoimmune encephalomyelitis (EAE) is a very important model for the exploration of new treatment options. Within the CNS, it reflects several pathomorphological features of MS, such as perivascular immune cell infiltration, activation of microglia and astrocytes, all contributing to demyelination and axonal loss. Relapsing EAE (R-EAE) was induced in female SJL/J mice using proteolipid protein peptide (PLP 139-151) [36,37], which classically peaked after 15 days and presented a maximal clinical score of 4.0 [27,38,39]. As compound **7i**’s effects on behavioral tests are visible at a concentration range of 0.1 to 1 mg/kg, the treatment was administrated at 0.5 and 1 mg/kg in EAE mice. Daily injections were performed for 15 days when the animals reached a score 2 (i.e., tail atony and/or clumsy gait). EAE disease course was followed for 80 days in order to observe the second relapse. Daily scoring of clinical signs in a blinded manner showed that EAE—vehicle (control) mice experienced onset at day 16 (D16 ± 1) after immunization (Figure 5A,B) and presented a clinical score ≥ 2 for 8 days (first relapse). Animals kept a score slightly above 2 for 30 days before experiencing a less intense second relapse.

Initially, compound **7i** was classically administrated intraperitoneally in a curative protocol. Compound **7i**’s effect was visible from the first days of treatment, with a more pronounced effect at a concentration of 1 mg/kg (Figure 5B). No second relapse was observed, regardless of the concentration tested (Figure 5A). When EAE disease course was followed for a longer time, EAE global evolution was significantly reduced when animals received the compound at 0.5 mg/kg. To confirm that the biological activity of compound **7i** required the presence of S1R, S1R binding sites were blocked with the S1R antagonist BD-1047, given intraperitoneally (10 mg/kg) 20 min before compound **7i** administration [27,40]. A similar clinical evolution was observed with the vehicle and BD-1047 pretreatment (Figure 5B). The same significant difference was observed when the EAE—**7i** group was compared to the EAE—vehicle and EAE—BD-1047 and **7i** group, showing that BD1047 blocked the effects of compound **7i** on EAE development, thus validating S1R specific mediated action. BD-1047 alone displayed no significant effect on EAE development [27].

In a second step, compound **7i** was administrated orally in the same curative protocol scheme. Very interestingly, a significant decrease in EAE course was observed after administration at 0.5 mg/kg (Figure 5C). This significant decrease in clinical intensity was observed as soon as the treatment was implemented (Figure 5D). Compound **7i**’s effect was maintained during the 15 days of administration, where animals reached grade 1. It is also important to note that orally treated animals do not exceed grade 2 during the entire disease course. No rebound effect was observed, as in i.p. administration (Figure 5C).

## 3. Discussion

The drug development process is typically divided into four major steps: discovery, proof of concept, preclinical development and clinical trial. In this paper, compound **7i** was used, whose synthesis and pharmacological evaluation has previously been investigated [28]. The implementation of a typical preclinical program demonstrates that, in addition to its excellent affinity for S1R, its high selective profile and its absence of cytotoxicity, compound **7i** has all the characteristics of an excellent drug candidate specifically targeting S1R. Its application in CNS disease, specifically in multiple sclerosis (MS), was demonstrated.

Knowledge of the possible drug metabolites that can be generated, as well as quantification and analysis of their individual activity, is important as it can potentially impede the future translation of therapeutic compounds into the clinic. To find out if there is an interspecies difference in metabolic stability profile, compound **7i** analysis was performed in mouse and human liver microsomes. The most important metabolite was identified as the demethylated compound, named compound **7i-deMe**. Compound **7i-deMe** also retains a good affinity for S1R with a high selectivity index. Preliminary ADME demonstrate that compounds **7i** and **7i-deMe** present solubility, permeability and plasma characteristics compatible with pharmacological development and that the route of administration of those compounds should be non-intravenous. Compounds have no impact on CYP inhibition in vitro, except CYP2D6. It was then decided to evaluate the potential of compound **7i** for drug–drug interaction (DDI). This step was taken early on in the study because, in the case of chronic neurodegenerative diseases, patients are likely to be prescribed medications to manage blood pressure, cardiovascular disease, joint inflammation, diabetes and other conditions associated with aging. Identifying the specific transporter for which a new compound is an inhibitor or a substrate improves drug bioavailability and efficacy, but also helps prevent adverse effects. Particular interest has been shown to BCRP and P-gp, expressed at the BBB. No drug transporter inhibition was observed, except that OCT1 and OCT2 were inhibited by compound **7i-deMe**. However, compound **7i** conversion into compound **7i-deMe** allows it to maintain part of its biological activity. As P-gp is crucial for absorption through active transport and CYP induction can affect drug efficacy through reducing plasma half-life, or drug toxicity if elevated levels of toxic metabolites are formed, assessment of those parameters was added to the analysis. The results obtained demonstrate that compound **7i** has all the necessary features to continue preclinical program development. Safety pharmacology and toxicology studies have also been undertaken. In fact, the potassium channel hERG is essential for normal electrical activity in the heart, and arrhythmia can be induced by a blockage of hERG by a surprisingly diverse group of drugs [35]. Given the similarity of reported S1R pharmacophore models to those postulated for hERG [41], and the direct interaction between S1R and hERG [42], compound **7i** was shown to inhibit hERG in vitro with an IC_50_ of 1 µM. As hERG channel blockers can cause long QT syndrome, which results in an increased risk of patients developing “torsades de pointes,” a fatal ventricular arrhythmia, the effect of systemic administration of compound **7i** on ECG interval duration, and specifically QT interval duration, was analyzed. It has been demonstrated that compound **7i** does not alter heart rate, nor PR or QRS duration in vivo. No prolongation of the QT interval was observed, leading to the conclusion that compound **7i** does not induce any pro-arrhythmic effect and confirming that development can be continued.

To complete this first phase of evaluation, cell cytotoxicity was classically analyzed by cell number counting. S1R is located at the MAM and is implicated in Ca^2+^ signaling between ER and mitochondria [13,14]. After activation, S1R also modulates several receptors, ion channels, transporters and enzymes localized at the plasma membrane [17,18]. No impact from compound **7i** was observed on intracellular free calcium, nuclear size, membrane permeability or mitochondrial membrane potential, certifying the absence of cytotoxic adverse effects. Furthermore, standard procedures established to analyze genetic toxicology for small molecules were used. Ames tests and induced chromosome damage (micronucleus assays) were performed. Finally, attention was focused on immunotoxicology that can manifest in a variety of ways, with one of the most prominent effects being immunomodulation and immunosuppression. Immunosuppressive agents cause a significant alteration in the body’s immune system, which enables opportunistic infections and malignancies [43]. Immunosuppressants decrease immunosurveillance, causing a subsequent lack of ability to fight infections. In MS, increased patient vigilance has been encouraged after several incidents of progressive multifocal leukoencephalopathy (PML) were reported in Natalizumab patients. Conversely, some drugs, specifically those used in cancer therapy, can promote the activation and expansion of the immune system and induce a wide range of immune-related adverse effects [44]. The S1R agonist 1(S), containing the tetra-hydroisoquinoline-hydantoin structure, was shown to decrease the magnitude of inflammation. The effect was associated with an increase in the proportion of B-cell subsets and regulatory T cells in the spleen and cervical lymph nodes [27]. Very interestingly, compound **7i** has no depleting or enhancing effect on several human immune cell types, including B, T, Th17 and regulatory T lymphocytes. Activated CD4, activated CD8 and natural regulatory T cells also retain their sensitive capacities and their ability to proliferate after stimulation, which confirms the safety of compound **7i**.

CNS-targeted diseases are unanimously considered to be one of the most important and difficult therapeutic challenges of our time. Accumulating evidence highlights the preclinical efficacy of selective drugs targeting S1R that act as agonists. Compounds showing S1R binding could also be allosteric modulators that have no pharmacological activity by themselves. Establishing the characterization of the activity of compound **7i** was crucial at this stage of the development phase. Evaluation of its capacity was firstly analyzed in vitro regarding S1R–BiP dissociation. The activity was then confirmed in vivo via the use of validated behavioral tests. Therefore, we were able to confirm that function of compound **7i** as a pharmacological agonist able to elicit a physiological response. The use of concentrations ranging from 0.1 to 5 mg/kg made it possible to highlight the bi-phasic bell-shaped dose–response curve, a common feature of S1R agonists [15] which was originally introduced through the notion of hormesis, defined as paradoxically beneficial effects seen with low doses and less beneficial effects at higher doses [45]. Compound **7i**’s maximum effects were visible at 0.5 to 1 mg/kg. It is important to note that S1R oligomerization states may also be important in regulating its functions [46]. S1R exists as dimers, tetramers, hexamers, octamers and perhaps even higher order oligomers. Oligomeric states of S1R could be stabilized by ligands [47]. The monomer form has been shown to bind to protein partners at the plasma membrane as a functional unit [48]. These data further enabled us to begin our in vivo preclinical studies. As the ultimate goal of pharmacological development is therapeutic establishment, pharmacokinetics (PK) parameters were evaluated for p.o. administration. Diffusion in the brain and the [brain]/[plasma] ratio demonstrated that compound **7i** exhibits excellent features for CNS diseases at nanomolar concentration. A comparison with the data from PRE-084, the prototypical drug used to uncover the potential therapeutic benefits of S1R [49], enables us to highlight that compound **7i** has all the qualities necessary to serve as a promising S1R-targeted drug [50].

Current MS treatments demonstrate high success in limiting new relapses and the accumulation of focal lesions; however, reparative approaches are still needed. Remyelinating treatment, as well as neuroprotective strategies, may lead to the reversal of neurological deficits. It is pivotal to highlight that development of oral drugs with sufficient bioavailability remains the forefront of preclinical research. EAE is an induced inflammatory disease of the CNS which follows the induction of immune response against CNS-specific antigens. A number of different models have been developed that can mimic certain stages of the MS disease; these commonly used models have directly contributed towards the development of a number of first-line MS treatments [37,51]. Compound **7i** was tested in a curative approach, for 15 days, in PLP-induced disease in SJL/J mice. This experimental chronic model offers a unique and interesting relapsing remitting course (R-EAE) comparable to the most common form of MS. Spinal cord demyelination and axonal damage are pathological markers of this model [37]. Treatment was therefore implemented during clinical disease, when animals reached clinical grade 2 (i.e., tail atony and/or clumsy gait). The compound was administrated at 0.5 and 1 mg/kg to induce an optimal effect, based on behavioral tests showing agonist activity and previous data from the laboratory [27]. EAE disease course was followed for 80 days in order to observe the emblematic acute (first relapse) and chronic (second relapse) phases of this R-EAE model. Decreases in EAE course were observed when compound **7i** was classically administrated intraperitoneally. As in behavioral tests, a bi-phasic bell-shaped dose–response curve was observed, with a greater significant effect on EAE global evolution at the lowest concentration. No second relapse was observed regardless of the concentration tested. The involvement of S1R was validated by using BD-1047 to saturate S1R binding sites before compound **7i** administration. Very interestingly, a significant decrease in EAE clinical intensity was also observed after oral administration of compound **7i** at 0.5 mg/kg, with a more pronounced effect as soon as the treatment was implemented (first relapse). Disease progression was stopped at clinical grade 2 and the action of compound **7i** was maintained at that time. Despite the interruption of the treatment, no rebound effect was observed.

As S1R controls ER stress and inflammatory processes in the cells, S1R agonists have been tested for a long time in preclinical studies and clinical trials for neuroprotection in neurodegenerative diseases, characterized by the progressive dysfunction of the structure and function of neuronal and glial cells and their network in the CNS. Many drugs interact with S1R and behave as agonists but are not selective. In the last few decades, S1R has emerged as a promising new target in MS. Eliprodil, an NMDA receptor antagonist with S1R affinity, has been shown to promote myelination in neuron–oligodendrocytes cocultures [52]. Dextrometorphan, an NMDA receptor antagonist and S1R agonist, demonstrated protective effects at low doses in EAE [53]. Additionally to dextometorphan, the mixed S1R and muscarinic receptor agonist ANAVEX2-73 (Blarcamesine), can also provide protection for oligodendrocytes and oligodendrocytes precursors and promote reparative therapy in MS [54]. Interestingly, the selective S1R agonist RC-33 demonstrated neurite elongation promotion in a rat dorsal root ganglia experimental cellular model, supporting the potential of S1R agonists in MS treatment [24]. Finally, our laboratory demonstrated that a single i.p. injection of the S1R agonist 1(S), containing the tetrahydroisoquinoline-hydantoin structure, decreased the clinical progression of EAE disease and prevented mononuclear cell accumulation and demyelination in the brain and spinal cord [27]. Nevertheless, compound 1(S) has only moderate metabolic stability. In this study, we thus decided to use compound **7i**, a simple benzamide-derived compound with excellent nanomolar affinity for S1R, selectivity for S2R, no cytotoxicity and good metabolic stability. It is important to note that extensive pharmacological profiling against 40 receptors relevant to CNS disease showed that it has a highly selective profile [28]. We have demonstrated that compound **7i** presents ADME properties, safety pharmacology and toxicology characteristics, as well as agonist capacity that makes it an excellent drug candidate for targeting CNS diseases. In an experimental model of MS, compound **7i** was successfully administrated orally in a curative procedure. Bell-shaped response, a common feature of S1R agonists, was observed during behavioral tests as well as in EAE, with an optimum effect at the lowest dose. At the molecular level, S1R can indeed be found in monomeric and oligomeric forms in physiological conditions, and it has been shown that agonists favor monomers and/or dimers while antagonists favor tetramers, hexamers, octamers and perhaps even higher order oligomers [17,48]. Dimer and monomer forms may represent the functional active forms, while the oligomers of S1R may serve as a reservoir for the active forms. At low doses, agonists activate S1R by binding to monomers and dimers who will then directly interact with other partner proteins, carrying out S1R chaperone activity. The oligomer reservoir allows the amount of active forms to be kept constant. At higher doses, agonists also interact with tetramers/hexamers/octamers, thus favoring an antagonist state that will lead to a decrease in global S1R response [15]. It should also be hypothesized that the bi-phasic dose-dependent effect observed could have an impact at the cellular dynamics level. It could indeed be that a low dose of the agonist facilitates S1R action on MAM, whereas a high dose of the agonist impacts the action of S1R on more distant cell compartments [15].

Altogether, these findings confirm that S1R represents a promising target for the development of affordable drugs against MS. S1R agonists may also be useful for adjunct treatment of MS and/or further development in progressive MS due to their manifold properties. The results obtained with compound **7i** confirm the positive role of S1R-selective benzamide-derived agonist compounds in the modulation of MS. This new drug candidate presents a moderate risk regarding its use in clinical trials. However, special attention should be paid to defining the optimum therapeutic window and the best concentration for use since S1R dose–response activity is bell-shaped for all drugs and blocked at high doses [15]. During clinical development, it will be important to keep in mind that lower doses have to be investigated as a priority. This most effective dose can only be found by relying on effective biomarkers [15,45]. It should also be interesting to analyze compound **7i**’s mode of action (MOA) given that boosting S1R activity leads to intracellular calcium homeostasis restoration, the stabilization of mitochondrial physiology and cellular adaptive response facilitation [16]. More precisely, calcium dynamics, as well as oxidative stress, should be studied under pathological conditions, particularly in oligodendrocytes and neurons. Neuroinflammation should also be studied since immune cell and cytokine impact are localized in the CNS. In the broader context of other CNS-targeted diseases, the impact of S1R modulation by compound **7i** on unfolded protein response, as well as on autophagy, could also be analyzed.

## 4. Materials and Methods

### 4.1. Chemical

Compound **7i** was synthesized according to Donnier-Maréchal et al. [28].

### 4.2. In Vitro Metabolic Stability and Metabolite Identification

Metabolic stability analysis (mouse and human liver microsomes) was performed by Eurofins Cerep (Poitiers, France) at 10 µM as described in [55]. Results were obtained at 15, 30, 45 and 60 min. Results were expressed as percent of the parent compound remaining, and calculated by comparing the peak area of the compound at the time point relative to that at time 0. The half-life (t_1/2_) and the apparent intrinsic clearance (CL_int_) were evaluated.

Metabolite identification was performed by HPLC-High Resolution mass spectrometry (MS). Chromatographic separation was performed on an Accela UPLC system (Thermo Fisher Scientific Inc. Waltham, MA, USA) equipped with an ACE 3 C18-AR column (100 × 2.1 mm, particle size 3 μm; AIT, Cormeilles-en-Parisis, France) preceded by a guard column (same stationary phase). The elution was performed with a gradient mode using water (solvent A) and ACN (solvent B), all acidified with 0.1% formic acid. The linear gradient elution program was as follows: 10% of B for 2 min; linear increase from 10 to 90% of solvent B for 20 min; 1 min at 90% of B; and 2 min of re-equilibration with 10% of B for a total run of 25 min. The LC flow rate was 400 µL/min and the injection volume was 10 μL. The column oven temperature was 30 °C and the sample tray temperature 4 °C. Eluted compounds were detected in positive mode by a full mass scan (*m*/*z* 200 to 500) using a Orbitrap Mass Spectrometer Exactive (Thermo Fisher Scientific Inc. Waltham, MA, USA) equipped with a heated electrospray ionization source (HESI-II). Instrument parameters were as follows: sheath gas 35, auxiliary gas 10 (both arbitrary units), spray voltage 2.4 kV, capillary temperature 275 °C, capillary voltage 67.5 V, tube lens voltage 120 V, skimmer voltage 24 V and source temperature 350 °C. Mass spectra were recorded at an ultra-high resolution (100,000). Instrument control, data acquisition and processing were performed using the associated Thermo Scientific software package (Xcalibur v.2.2 and Exactive v1.1).

### 4.3. Assay for Binding to Sigma Receptors

The binding assays were performed by Eurofins Cerep (Poitiers, France) according to Ganapathy [56]. Briefly, S1R)binding assay was carried out by incubating Jurkat cell membranes (10–20 mg protein per tube) with [^3^H] (+)-pentazocine (15 nM) and a range of concentrations of test compounds at 37 °C for 2 h in 5 mM Tris HCl buffer (pH = 7.4). The S2R binding assay was performed by incubating Jurkat cell membranes (10–20 mg protein per tube) with [^3^H]-DTG (25 nM) in the presence of (+)-pentazocine (1 mM), to saturate S1R, and a range of concentrations of test compounds at room temperature for 1 h in 5 mM Tris HCl buffer (pH = 7.4). Bound radioactivity was measured using liquid scintillation counting. Nonspecific binding was determined in both assays, under similar conditions, as remaining in the presence of 10 mM unlabeled haloperidol. Inhibition constants (Ki) were calculated from the IC_50_ values according to the method of Cheng and Prusoff [57].

### 4.4. Physicochemical Properties, Absorption, Distribution and Toxicity

Toxicity analysis was performed in the human neuroblastoma cell line (SH-SY5Y), which was cultured in Dulbecco’s Modified Eagle Medium (DMEM, Gibco) supplemented with 2 mM L-glutamine (Invitrogen, Thermo Fisher Scientific Inc. Waltham, MA, USA), 100 mg/mL streptomycin (Invitrogen), 100 IU/mL penicillin (Invitrogen), 1 mM non-essential amino acids (Invitrogen) and 10% (*v*/*v*) heat inactivated fetal bovine serum (Sigma Aldrich, Burlington, MA, USA) and grown at 37 °C in a humidified incubator with 5% CO_2_. Cells were starved for 24 h to obtain synchronous cultures and were then incubated in culture medium that contained various concentrations of test compounds (100, 50, 10, 5, 1, 0.5, 0.1, 0.05 mM), each dissolved in less than 0.1% DMSO. After 72 h of incubation, cell growth was estimated by the colorimetric MTT assay (CellTiter 96^®^ aqueous one solution cell proliferation assay-MTS, Promega, Madison, WI, USA) according to the manufacturer’s protocol. Absorbance was read at 490 nm and results were expressed as % from the control condition considered as 100%.

Standardized in vitro ADME experiments were performed by Eurofins Cerep (Poitiers, France). Solubility assays were performed in PBS at pH 7.4, but also in simulated gastric fluid (SGF) and intestinal fluid (SIF), through the classical shake-flask method according to Lipinski et al. using HPLC-UV/VIS technology [58]. Results were expressed in µM.

The compound was classically evaluated at 10 µM for all the ADME analyses. Bidirectional permeability (apical A to basolateral B pH 6.5/7.4 and basolateral B to apical A pH 7.4/6.5) was assessed in the epithelial colorectal adenocarcinoma cell line (Caco-2 cell line) [59]. Results were expressed as × 10^−6^cm/s (Papp) and as % recovery. Efflux ratio, i.e., Papp(B-A)/Papp(A-B), was calculated. Plasma protein binding was analyzed by HPLC-MS/MS after 4-h incubation at 37 °C, as described by Banker et al. [60]. Concentration was evaluated by LC/MS-MS. CYP inhibition was performed in human liver microsomes as described in [61]. Analysis of the respective metabolites. was performed by HPLC-MS/MS. Transporter inhibition analysis was also performed in overexpressing cell lines. Respective metabolites for each specific transporter substrate were analyzed by fluorometry or scintillation counting and % inhibition of control values were calculated [62,63,64,65,66,67,68,69,70,71]. P-glycoprotein transporter substrate assessment was performed by bidirectional permeability analysis (apical A to basolateral B pH 7.4/7.4 and basolateral B to apical A pH 7.4/7.4) in the Caco-2 cell line with and without verapamil, as described in [59]. Results were expressed as ×10^−6^ cm/s (Papp) and as % recovery. The efflux ratio, i.e., Papp(B-A)/Papp(A-B), was calculated. CYP450 induction characterization was performed on human hepatocytes. The mRNA levels of 7 different CYPs were analyzed by qPCR, as described in [72]. The compound was evaluated at 1, 10 and 100 µM. Fold-induction values were determined and compared to cut-off values, as defined for each CYP isozyme in each hepatocyte lot.

Plasma stability analyses were performed by M2SV (Lille, France). Incubations were performed in duplicate. The plasma (male mouse plasma CD-1, lithium-heparinized, from Sera Laboratories International Ltd. BioIVT, West Sussex, UK) was pre-incubated for 10 min at 37 °C before addition of the compound to a final concentration of 10 µM (0,1% DMSO). At the defined time points (0, 60, 1440 and 2880 min), fractions from each tube were transferred to fresh tubes containing cold CH_3_CN and an internal standard (Propanolol at 1 µM). After vigorous agitation and centrifugation (10 min at 10,000 rpm), the supernatants were analyzed. The degradation half-life (t_1/2_) values were calculated in minutes and hours via non-linear regression analysis using Microsoft^®^ XLfit^®^ Excel add-in from IDBS Ltd, Woking, UK.

### 4.5. Toxicology

Cardiotoxicity analysis was performed by Eurofins Cerep (Poitiers, France) according to [73]. hERG-CHO overexpressing cells were used to perform automated patch-clamping. Compound **7i** was evaluated at 0.1, 1 and 10 µM. Activities were expressed as % inhibition of tail current.

Cytotoxicity analysis was performed by Eurofins Cerep (Poitiers, France) according to [74]. Compound **7i** was evaluated at 10 µM in the HepG2 cell line. Cell number, nuclear size, mitochondrial membrane potential, intracellular free calcium and membrane permeability were analyzed. % inhibition or % increase were calculated.

The potential mutagenic effects of compound **7i** were evaluated by the Pasteur Institute’s genetic toxicology laboratory (Lille, France) on a bacterial model using Ames tests [75]. Six strains were used, with and without metabolic activation by the S9 fraction. Colonies were counted and an induction ratio was calculated. The potential genotoxic effects of compound **7i** were evaluated in vitro using the mammalian cell micronucleus test on TK6 human cells, with and without metabolic activation by the S9 fraction [76,77]. The frequency of micronucleated (MN) cells was determined out of at least 2000 mononucleated cells. Cell viability was performed under the same conditions, using a colorimetric MTT assay, to validate that cytotoxicity did not exceed 50%.

Immunotoxicity analysis was performed by Immune Insight CRO (Lille, France) on human peripheral blood mononuclear cells (hPBMCs) from six individual healthy donors. hPBMCs were either non-stimulated (basal) or stimulated using *E. coli* lipopolysaccharide (LPS, Sigma Aldrich, St. Louis, MO, USA) at 100 ng/mL. hPBMCs were activated with plate-bound anti-CD3 (10 ng/mL) monoclonal antibody (mAb) and incubated at 37 °C for 2 h before the culture and soluble anti-CD28 (10 ng/mL) mAb (Clinisciences, Montrouge, France) were added. Compound **7i** was tested at 10 µM. The quality of hPBMC activation was controlled using cytometric analysis based on CD25 staining and proliferation assays based on ^3^H-thymidine incorporation [78]. Immune cell subpopulations were immunophenotyped among hPBMCs by flow cytometry on an Attune NxT (Thermofisher Scientific Inc. Waltham, MA, USA). Analyses were performed at 48 h post treatment. The expression of B, CD4, CD8, Th17 and Treg lymphocyte surface antigens was tested using monoclonal mouse anti-human Ab (Miltenyi Biotec SAS, Paris, France) (Table 9). FoxP3-APC intracellular staining was achieved with FoxP3 staining buffer (Miltenyi Biotech SAS, Paris, France). Phenotype analysis was performed by gating CD4 (CD3+, CD4+), activated CD4 (CD3+, CD4+, CD25+, CD127+, FoxP3+), helper (CD3+, CD4+, CCR6−), CD8 (CD3+, CD8+), activated CD8 (CD3+, CD8+, CD25+) T lymphocytes, natural T regulatory cells (CD3+, CD4+, CD25Hi, CD127Lo/−, FoxP3+), Th17 (CD3+, CD4+, CCR6+) lymphocytes and B (CD3−, CD19+) lymphocytes; all lymphocytes were discriminated on live cells (+ live/dead marker).

### 4.6. In Vitro Evaluation of S1R Functionality

The activity of compound **7i** was evaluated in regard to its ability to dissociate the S1R–BiP complex. The S1R–BiP dissociation assay was performed by Amylgen (Montferrier-sur-Lez, France) as described in [13,79]. Briefly, CHO cells were treated with test compounds for 30 min (37 °C). Crosslinking was performed with dithiobis succinimidyl propionate (DSP; Thermoscientific, Waltham, MA, USA). Lysis was performed after incubation for 15 min on ice. After centrifugation, supernatants were incubated overnight (4 °C) with the S1R antibody (Abcam, Cambridge, UK). Lysates were incubated with Sepharose Protein-A (Invitrogen). After the first centrifugation, the pellet was resuspended in radioimmunoprecipitation assay (RIPA) buffer. After a second centrifugation, the pellet was resuspended in 2× sample buffer/bMCE buffer. After the third centrifugation, supernatants were analyzed for BiP immunoreactivity (SEC343Mu ELISA, USCNK Life Sciences, Wuhan, China).

### 4.7. In Vivo Evaluation of S1R Functionality

The activity of compound **7i** was evaluated in regard to its ability to reverse dizocilpine-induced learning deficits. Two behavioral tests were performed by Amylgen (Montferrier-sur-Lez, France) on male Swiss mice (aged 6–9 weeks). All compounds (**7i** and controls) were intraperitoneally injected.

Animals were tested for spontaneous alternation performance in the Y-maze, an index of spatial working memory [80,81]. Made of grey polyvinylchloride, the Y-maze has an arm which is 40 cm long, 13 cm high and 3 cm wide at the bottom and 10 cm wide at the top, converging at an equal angle. Each mouse was placed at the end of one arm and allowed to move freely through the maze during an 8-min session. The series of arm entries, including possible returns into the same arm, were checked visually. An alternation was defined as entries into all three arms on consecutive occasions. The number of maximum alternations was therefore the total number of arm entries minus two, and the percentage of alternation was calculated as: (actual alternations/maximum alternations) × 100. Parameters included the percentage of alternation (memory index) and the total number of arm entries (exploration index). Animals showing extreme behavior (alternation < 20% or >90% or number of arm entries < 10) are usually discarded from the calculations. Animals were also tested using the step-through passive avoidance test to measure non-spatial/contextual long-term memory [81] by using a grid-floor 2-compartment box separated by a guillotine door that included: (1) an illuminated compartment (60 W lamp, 40 cm above) with white polyvinylchloride walls and a transparent cover (15 × 20 × 15 cm) and (2) a darkened compartment with black polyvinylchloride walls and cover (15 × 20 × 15 cm). Scrambled footshocks (0.3 mA for 3 s) were delivered to the grid floor using a shock generator scrambler (Lafayette Instruments, Lafayette, LA, USA). The guillotine door was initially closed during the training session. Each mouse was placed into the white compartment. After 5 s, the door was raised. When the mouse entered the dark compartment and placed all its paws on the grid floor, the door was closed and the footshock delivered for 3 s. Step-through latency (the latency in time spent to enter the dark compartment) and the number of vocalizations were recorded. The retention test was carried out after 24 h. Each mouse was again placed into the white compartment. After 5 s, the door was opened. Step-through latency was recorded up to 300 s. When either the mouse entered the dark compartment or 300 s had elapsed (they were therefore manually placed in it), escape latency (latency to exit from the dark compartment) was recorded with a value of up to 300 s.

Animals were used at day 1 in the Y-maze test and at days 2 and 3 in the passive avoidance test, with training at day 2 and retention at day 3. Compound **7i** (0.1, 0.5 and 1 mg/kg) was administered 10 min before dizocilpine (0.15 mg/kg) or vehicle control. Dizocilpine or vehicle control was administered 20 min before the Y-maze test session on day 1 and 20 min before the passive avoidance training session on day 2. Drugs were not injected before the retention session on day 3. The anti-amnesic S1R agonist activity of compound **7i** was validated using a pre-treatment with the reference S1R antagonist NE-100 (0.15 mg/kg). Compound **7i** and/or NE-100 were administered 10 min before dizocilpine or vehicle control.

### 4.8. Phamacokinetic Analyses

A pharmacokinetic (PK) study was performed by Eurofins Cerep (Poitiers, France) in male C57BL/6 mice (20–30 g) following i.v. and p.o. administrations of compound **7i** at 1 mg/kg.

The plasma and perfused brain samples were collected at 0, 30, 60, 120, 240 and 280 min after treatment administration (*n* = 3 animal/time point). Animals were terminated under inhalant CO_2_ euthanasia. Blood was collected via cardiac puncture. Aliquots were collected in tubes coated with lithium heparin and centrifuged. The plasma was harvested and kept frozen at −70 °C until further processing. Immediately following the collection of the blood sample, animals were perfused with heparinized saline (10 IU/mL). The brain was collected, rinsed with saline to remove residual blood, weighed and stored at −70 °C until further analysis.

Plasma samples were processed using acetonitrile precipitation and analyzed by LC-MS/MS. A plasma calibration curve was generated. Aliquots of drug-free plasma were spiked with compound **7i** at the specified concentration levels. The spiked plasma samples were processed together with the unknown plasma samples using the same procedure.

Each brain was homogenized for 10 s on ice in a proper volume of cold phosphate-buffered saline (PBS) at pH 7.4. The homogenate was centrifuged at 5400× *g* for 15 min at 4 °C. Supernatants were subsequently processed using acetonitrile precipitation and analyzed by LC-MS/MS. A brain calibration curve was generated. Aliquots of drug-free brain homogenate were spiked with compound **7i** at the specified concentration levels. The spiked brain homogenate samples were processed together with the unknown brain homogenate samples using the same procedure.

Concentrations of compound **7i** in the unknown plasma and brain samples were determined using the respective calibration curve. The reportable linear range of the assay was determined, along with the lower limit of quantitation (LLQ).

The exposure levels of compound **7i** in the plasma and brain samples as determined by LC-MS/MS were reported (ng/mL). The [brain]/[plasma] ratios were calculated.

Pharmacokinetic modeling was performed using the non-compartmental method based on statistical moments. In this method, exchanges between kinetic spaces are expressed in time. The curves were obtained using R software (Package ggplot2, v3.3.6).

### 4.9. Animals

Animals were fed with ad libitum access to food and water in compliance with the European standards for the care and use of laboratory animals, and the experiments conducted in this study were authorized by the French Direction of Veterinary Services with the approved registration numbers. Efforts were made to minimize the number of animals used and their suffering. All studies involving animals are reported in accordance with the ARRIVE guidelines for reporting experiments involving animals [82,83], and more particularly for those using experimental autoimmune encephalomyelitis (EAE) [84].

Mice were purchased from Janvier (Le Genest-St-Isle, France) and bred under conventional barrier protection at the Lille Pasteur Institute (France) and at the Hospitalo-Universitary Department of Experimental Research of Lille (DHURE, France).

### 4.10. Pro-Arrhythmic Effects

Electrocardiograms were recorded on C57BL/6 male mice (*n* = 2–3 animal/group) anesthetized with isoflurane (5% induction, 1.5% maintenance). Four electrodes were inserted subcutaneously in the mouse limbs. Electrical signals were recorded with a Powerlab (Ad instruments) and analyzed with LabChart Pro software (v8.1.19).

Heart rate and PR, QRS and QT intervals were measured as described in Supplementary Figure S3. We compared basal QT duration and its modification 3, 6, 10, 20 and 30 min after i.p. administration of compound **7i**, at concentrations of 0.5 mg/kg, 1 mg/kg, 5 mg/kg and 10 mg/kg, or its vehicle. Quinidine (Quinimax, Sanofi Aventis, Gentilly, France) is an anti-arrhythmic drug known to prolong QT duration and was used as a positive control (10 mg/kg, 100 mg/kg).

### 4.11. EAE Induction and Evaluation

The method of EAE induction has been described previously [39]. Randomized 9-week-old female SJL/J mice were inoculated subcutaneously (s.c.) in the neck with an emulsion containing 100 μg of myelin proteolipid protein (PLP)139–151 peptide and an equal volume of complete Freund’s adjuvant (CFA) containing 4 mg/mL of heat-inactivated Mycobacterium tuberculosis H37RA (Difco Laboratories, Detroit, MI, USA) on day 0 (D0). Additionally, mice received 0.3 μg of *Bordetella pertussis* toxin (BPT; Sigma-Aldrich, Saint Louis, MI, USA) intraperitoneally on D0 and D3. Control animals received administration of saline solution (EAE—vehicle mice).

Three different treatment groups (EAE—vehicle, EAE—**7i** 0.5 mg/kg and EAE—**7i** 1 mg/kg) were used, with 5–6 animals per treatment group. Compound **7i** was intraperitoneally or orally administered when mice presented clinical grade 2. The involvement of S1R in the beneficial effects of compound **7i** was validated by S1R antagonist BD1047 (Tocris Biotechne, Bristol, UK) administration (10 mg/kg, i.p.) 20 min before compound **7i** injection. Body weight and clinical signs of EAE were monitored daily. The severity of clinical symptoms was scored based on the standard neurological scoring system for EAE as follows: grade 0, no disease; grade 1, moderate tail hypotonia and/or slightly clumsy gait; grade 2, tail atony and/or clumsy gait; grade 3, severe hind limb paresis; grade 4, paraplegia; grade 5, tetraplegia; and grade 6, dead. Clinical evaluations were performed in a blinded manner, without knowledge of the treatments. Based on the clinical score data, EAE was characterized using the following parameters: cumulative disease index (CDI), mean score at disease peak (D16 ± 1) and mean score during treatment administration. The CDI was calculated as the sum of the daily clinical scores for each mouse. Disease peak refers to the first day of maximal clinical scores for EAE—vehicle mice.

Mice showed no apparent toxic side effects from any of the treatment protocols. Animals that reached severe hind limb paresis (clinical grade 3) were isolated and hydration and food access were facilitated. Mice were deeply anaesthetized with an i.p. injection of pentobarbital. Serum samples were prepared from peripheral blood obtained by cardiac puncture. Active immunizations were confirmed by measuring the anti-PLP139–151 IgG antibody (Ab), as previously described [85].

### 4.12. Statistical Analysis

For each type of experiment, group sizes are given in the figure legends and data have been presented as the mean ± standard error of the mean (SEM). No data outliers were excluded.

For immunotoxicity analysis, one-way analysis of variance (ANOVA) was performed, followed by pairwise comparisons versus basal vehicle condition using Wilcoxon–Mann–Whitney U tests. Statistical analysis was performed using one-way ANOVA, followed by the Dunnett’s post-hoc multiple comparison test for S1R–BiP association. For in vivo evaluation of S1R functionality, statistical analysis was performed using two-way ANOVA analysis followed by Dunn’s post-hoc multiple comparison test for assessment of spontaneous alternation performance. Passive avoidance latencies were analyzed using Kruskal–Wallis non-parametric ANOVA followed by Dunn’s post-hoc multiple comparison test. In vivo QT intervals were analyzed using Wilcoxon–Mann–Whitney U tests. For the comparison of EAE scores between groups, a two-way ANOVA was performed. Pairwise comparisons between the EAE—**7i** versus EAE—vehicle group were conducted using Wilcoxon–Mann–Whitney U tests.

Values of probability *p* < 0.05 were considered statistically significant. The level of significance was labeled as * *p* < 0.05, ** *p* < 0.01 or *** *p* < 0.001.

## 5. Patents

Some of the results presented in this article are detailed in the patent EP14305919.

## Figures and Tables

**Table 1:** Metabolic stability profiles. Microsomal stability of compounds **7i** and **7i-deMe** was assessed in mouse (mLM) and human (hLM) liver microsomes at 10 µM. Half-life (t_1/2_) and intrinsec clearance (CL_int_) were evaluated. % compound remaining after 1 h was determined.

		mLM			hLM		
		t_1/2_ (h)	CL_int_ (µL/min/pmol)	Compound Remaining (1 h, %)	t_1/2_ (h)	CL_int_ (µL/min/pmol)	Compound Remaining (1 h, %)
**7i**	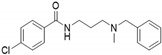	1.2	<115	54	1.9	<115	66
**7i-deMe**	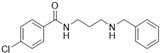	3.9	<115	80	10.0	<115	83

**Table 2:** S1R and S2R affinities and the cytotoxicity of compounds **7i** [28] and **7i-deMe**.

	Ki			Inhibition
	S1R (nM)	S2R (nM)	Ratio S2R/S1R	SH-SY5Y (% at 100 µM)
**7i** [28]	3.2	190	60	28
**7i-deMe**	23	>1000	>500	77

**Table 3:** Preliminary ADME study of compound **7i** and major metabolite **7i-deMe**. Solubility was analyzed in PBS, simulated Gastric fluid (SGF) and simulated intestinal fluid (SIF). Efflux ratio (e-ratio), plasma half-life (t_1/2_) and plasma protein binding (PPB) were calculated. CYP and drug transporter inhibition, P-gp substrate and CYP induction were analyzed. CYP induction analysis was carried out on three donors whose individual cut-offs are noted in brackets. Analyses were performed at 10 µM. Results are expressed as mean (*n* = 2).

	7i	7i-deMe
**Solubility (µM)**		
PBS_pH7.4_	185	187
SGF	193	197
SIF	188	196
**Permeability (×10^−6^ cm/s)**		
A/B _pH6.5/7.4_	29.1	31.1
B/A _pH6.5/7.4_	66.1	51.5
e-ratio	2.3	1.7
**Plasma t_½_ (h)**	25.9	23
**PPB (%)**	94	81
**CYPs inhibition (%)**		
CYP1A	2.1	9.4
CYP2B6	−4.3	15.9
CYP2C8	−9.2	−7.2
CYP2C9	−7.2	5.9
CYP2C19	36.5	18.4
CYP2D6	95.1	91.6
CYP3A	36.9	3.2
**Drug transporters inhibition (%)**		
**ABC family**		
P-gp	10.6	1.0
BCRP	8.3	−3.0
MRP1	1.9	−3.4
MRP2	−18.8	−11.5
MRP3	−0.3	−0.6
**SLC family**		
OATP1B1	12.0	8.6
OATP1B3	23.1	19.1
OAT1	−9.6	−6.2
OAT3	34.3	18.7
OCT1	41.2	64.6
OCT2	49.4	85.8
ABST	5.3	5.0
NTCP	−1.7	8.7
**P-gp substrate (×10^−6^ cm/s)**		
**PBS (×10^−6^ cm/s)**		
A/B _pH7.4/7.4_	45.8	
B/A _pH7.4/7.4_	11.7	
e-ratio	0.3	
**Verapamil (×10^−6^ cm/s)**		
A/B _pH7.4/7.4_	28.0	
B/A _pH7.4/7.4_	16.9	
e-ratio	0.6	
**CYPs induction (%)**		
**CYP1A2**		
#1 _(2)_	2.7	
#2 _(5)_	1.5	
#3 _(2)_	1.9	
**CYP2B6**		
#1 _(3)_	4.1	
#2 _(4)_	4.1	
#3 _(2)_	4.2	
**CYP3A4**		
#1 _(4)_	4.2	
#2 _(6)_	1.6	
#3 _(3)_	0.8	

**Table 4:** Compound **7i** cardiotoxicity and cytotoxicity analysis. Results are expressed as mean (*n* = 2).

	7i
**Cardiotoxicity**	
**hERG inhibition (mM)**	
IC_50_	1
**Cytotoxicity**	
**HepG2 (% at 10 µM)**	
Cell number	−3
Intracell free calcium	0
Nuclear size	9
Membrane permeability	0
Mitochondrial membrane potential	5

**Table 5:** Compound **7i** genotoxicity analysis using the Ames test. Five *Salmonella S. typhimurium* strains were used: TA1535, TA97a, TA98, TA100 and TA102 with (S9+) and without (S9−) external metabolic activation. The OECD 471 guideline was followed. Mean revertants/well of the technical triplicates in an experiment are shown. Positive controls (C+) are shown. (a) TA1535:MNNG 0.25; TA1537: 9-amino-acridine 1.56; TA98: 2-nitrofluorene 0.5; TA100: MNNG 0.25; TA102: Mitomycine 0.0625. (b) TA1535, TA1537, TA98, TA100:2-anthramine 0.5; TA102: Benzo(a)pyrene 4. Results are expressed as mean (*n* = 2).

		TA1535	TA1537	TA98	TA100	TA102
	Dose (µg/plate)	−S9	−S9	−S9	−S9	−S9
Positive control	(a)	85.9	8.4	23.1	13.5	6.0
Vehicle control	0	-	-	-	-	-
**7i**	40	1.3	0.9	1.1	0.9	0.7
		**+S9**	**+S9**	**+S9**	**+S9**	**+S9**
Positive control	(b)	26.9	27.6	36.0	6.0	5.7
Vehicle control	0	-	-	-	-	-
**7i**	40	0.9	1.1	1.1	0.9	1.0

**Table 6:** Compound **7i** genotoxicity analysis with a micronucleus (MN) in TK6 cells. Analysis was performed after 3-h treatment with DHA, rutin and α-tocopherol with (S9+) and without (S9−) metabolic activation and after 24 h without metabolic activation. MN per 103 nucleated cells and the relative survival rate (RS) of two independent experiments are shown. Results are expressed as mean (*n* = 2).

		3 h Short Treatment With 24 h Recovery Period						27 h Continuous Treatment		
		−S9			+S9			−S9		
		µg/mL	RS (%)	MN/2 × 10^3^ Cells	µg/mL	RS (%)	MN/2 × 10^3^ Cells	µg/mL	RS (%)	MN/2 × 10^3^ Cells
Positive control	**Mytomice C**	0.5	62.8	153.0				0.2	67.9	62.0
	**Griseofulvin**							5	70.5	63.0
	**Cyclophosphamide**				5	88.3	35.5			
Vehicle control		0	-	3.0	0	-	11.0	0	-	9.5
**7i**		275	80.0	8.0	275	103.7	9.0	275	70.1	6.0
		137.5	92.7	8.0	137.5	97.2	6.0	137.5	84.2	7.0
		68.78	94.1	6.0	68.75	106.1	10.5	68.75	86.6	1.0

**Table 7:** Pharmacokinetic parameters of compound **7i**. Compound **7i** was i.v. and p.o. administrated at 1 mg/kg. Analyses were performed in plasma and the brain. Maximal concentration (C_max_), time to reach C_max_ (t_max_) and concentration at 30 min (C_30_) were analyzed. The correct distribution of the time-points was validated using area under the curve (AUC) parameters (AUC _t_^∞^/AUC _∞_). Distribution volume (V_d_), total clearance (CL_T_), brain clearance (CL_brain_), half-life of elimination (t_1/2 el_), mean absorption time (MAT), bioavailability (F) and diffusion in the brain (D) were calculated. The [brain]/[plasma] ratio was calculated at 30 min. Data were obtained using a median value of *n* = 3 mice per time-point. * for simulated, NA for not available.

	i.v	p.o
**Plasma**		
t_max_ (min)	0	≤30
C_max_ (ng/mL) *	180	NA
C_30_ (ng/mL)	79.8	6.4
AUC _t_^∞^/AUC _∞_	0.9	6.1
V_d_ (mL/kg)	6.1	168.0
CL_T_ (mL/min)	3.8	74.9
t_1/2 el_ (min)	44.5	25.5
MAT (min)	0	16.5
F (%)	100	5
**Brain**		
t_max_ (min)	≤30	≤30
C_30_ (ng/mL)	234.3	20.7
AUC _t_^∞^/AUC _∞_	5.9	7.6
CL_brain_ (mL/min)	1.8	12.8
t_1/2 el_ (min)	96.4	78.9
D (%)	61.7	NA
**Brain/Plasma**	2.9	3.2

**Table 8:** In vivo QT interval analysis. QT intervals measured via ECG under isoflurane anesthesia in mice injected with compound **7i**, quinidine (positive control) or vehicle control. Data are presented as mean ± SEMof *n* = 2–3 mice per group. Differences between baseline QT intervals and QT intervals following i.p. injections were tested. * for *p* < 0.05 versus basal QT interval in the same group. Wilcoxon–Mann–Whitney U test.

		QT Interval					
			Time after Injection				
	Dose (mg/kg)	Basal	3 min	6 min	10 min	20 min	30 min
**Vehicle**		51 ± 2	46 ± 3	45 ± 4	56 ± 9	62 ± 7	63 ± 11
**7i**	0.5	49 ± 3	48 ± 4	46 ± 4	53 ± 3	59 ± 4	58 ± 3
	1.0	48 ± 1	45 ± 2	46 ± 2	>49 ± 1	>	
	>5.0	>46 ± 3	>43 ± 1	>42 ± 2	>43 ± 2	>	
	10.0	46 ± 1	43 ± 1	45 ± 3	49 ± 2	52 ± 1	54 ± 1
**Quinidine**	10.0	46 ± 2	50 ± 2	64 ± 6	65 ± 5 *		
	100.0	47 ± 4	58 ± 3	84 ± 7 *	76 ± 4 *		

**Table 9:** List of monoclonal antibodies used for flow cytometric analysis.

Product	Manufacturer	Reference Number
CD3 Antibody, anti-human APC-VIO^®^ 770	Miltenyi Biotec	130-113-136
CD4 Antibody, anti-human PerCP-Vio^®^ 700	Miltenyi Biotec	130-113-228
CD8 Antibody, anti-human VioGreen™	Miltenyi Biotec	130-110-684
CD25 Antibody, anti-human, Vio^®^ Bright B515	Miltenyi Biotec	130-115-536
CD127 Antibody, anti-human, PE-Vio^®^ 770	Miltenyi Biotec	130-113-415 130-113-412
CD19 Antibody, anti-human, PE-Vio^®^ 615	Miltenyi Biotec	130-114-522
CD196 (CCR6) Antibody, anti-human, PE	Miltenyi Biotec	130-120-458
FoxP3 Antibody, anti-human, APC	Miltenyi Biotec	130-125-580
Viobility 405/452 Fixable Dye	Miltenyi Biotec	130-110-205

## Data Availability

Not applicable.

## Supplementary Materials

The supporting information can be downloaded at: https://www.mdpi.com/article/10.3390/ijms231911893/s1.
