# Supplementary material for: Safe and Efficient Sigma1 Ligand: A Potential Drug Candidate for Multiple Sclerosis
Source: Int J Mol Sci. 2022 Oct 6;23(19):11893. doi: 10.3390/ijms231911893 (PMC9569529; doi:10.3390/ijms231911893)
Supplement: Supplementary file 1 [file ijms-23-11893-s001.zip › ijms-1938735-SM.pdf]

International Journal of
Molecular Sciences

MDPI

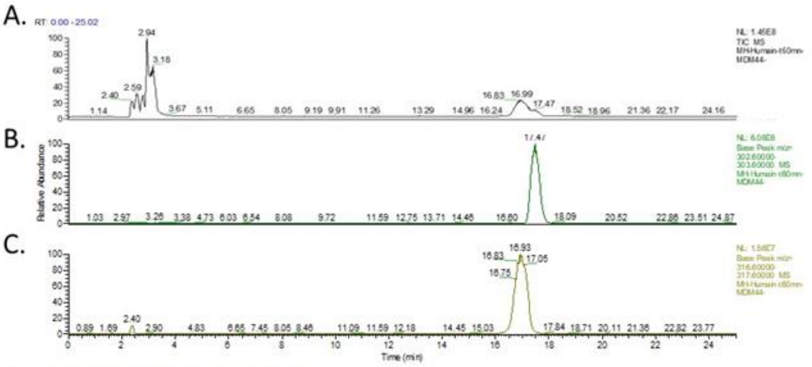

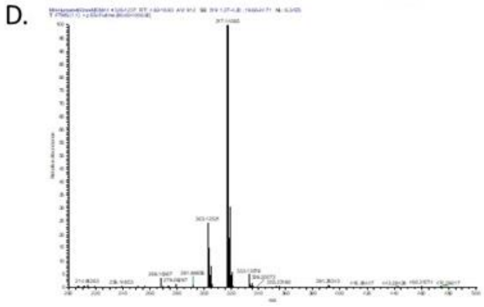

E.

	7i	7i-deMe
m/z	317.14086	303.12521
Intensity	3318764.3	863981.1
Relative	100	26.03
Theo. Mass	317.14152	303.12587
Delta (ppm)	-2.07	-2.18
RDB equiv.	8.5	8.5
Composition	$C_{18}H_{22}ON_2Cl$	$C_{17}H_{20}ON_2Cl$

Figure S1. Metabolite identification and profiling. Chromatographic separation of compound **7i** and its major metabolite (panel A) obtained from human liver microsomes after 1h incubation. Base peak of the demethylated compound **7i-deMe** (panel B) (m/z = 303.12521) and of the compound **7i** (panel C) (m/z = 317.14086). Mass spectra validation (panel D) and characteristics of the compounds (panel E).

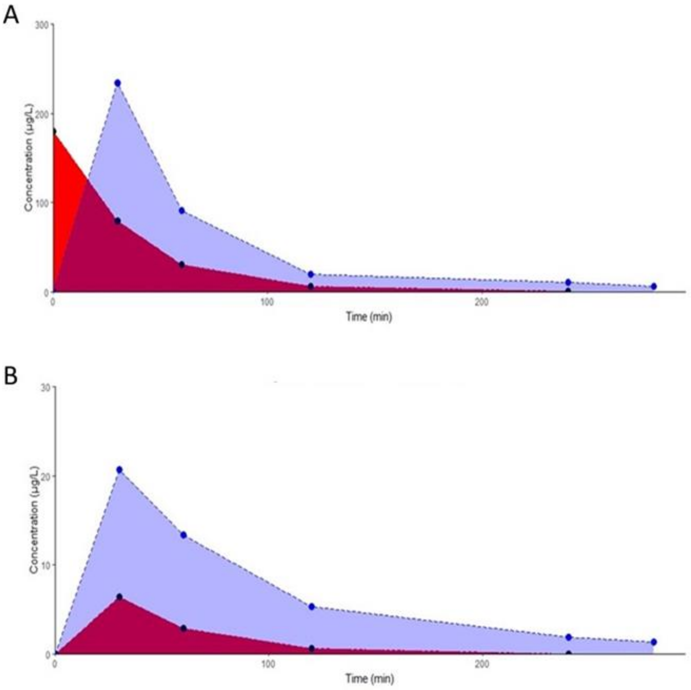

Figure S2. Compound 7i kinetics after i.v (panel A) and p.o (panel B) administration. Compound 7i was administrated at 1mg/kg. Analyses were performed in plasma (red) and brain homogenates (blue). Data are presented as median of n=3 mice per time-point. Simulations were performed to define time 0 to 30 min evolution.

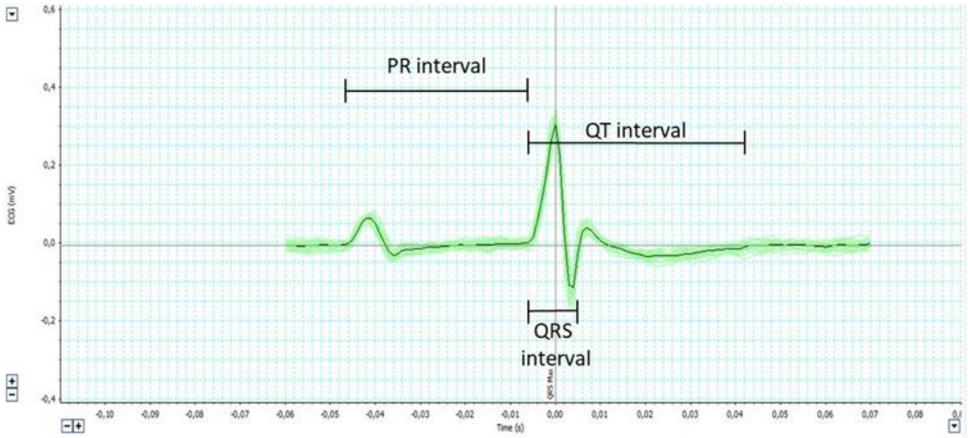

Figure S3. Normal ECG trace for single cardiac cycle.The PR interval represent the period extending from the beginning of the P wave until the beginning of the QRS complex. The QRS interval represent depolarization. The QT interval represents the time between initial depolarization and final repolarization.
